# Using Virtual Reality to Study Spatial Mapping and Threat Learning

**DOI:** 10.21203/rs.3.rs-3891586/v1

**Published:** 2024-04-03

**Authors:** Claire E. Marino, Pavel Rjabtsenkov, Caitlin Sharp, Zonia Ali, Evelyn Pineda, Shreya Bavdekar, Tanya Garg, Kendal Jordan, Mary Halvorsen, Carlos Aponte, Julie Blue, Xi Zhu, Benjamin-Suarez Jimenez

**Affiliations:** 1 Department of Neuroscience, University of Rochester School of Medicine and Dentistry, Rochester, NY 14642, USA; 2 Department of Psychology, University of Rochester, Rochester, NY 14627, USA; 3 Department of Psychiatry, Columbia University Irving Medical Center, New York, NY 10032, USA; 4 New York State Psychiatric Institute, New York, NY 10032, USA

## Abstract

Using spatial mapping processes to learn about threat and safety in an environment is crucial for survival. Research using conditioning paradigms has explored the effects of state (transient arousal) and trait anxiety (anxiety as an aspect of personality) on threat learning and acquisition. However, results are mixed, and little is known regarding why some individuals do not learn to discriminate between threat and safety during contextual conditioning. We used a virtual reality (VR) contextual threat conditioning paradigm to elucidate the effects of state and trait anxiety on contextual threat learning. 70 healthy participants (46 female) navigated and “picked” flowers in a VR environment. Flowers picked in the dangerous zone (half of the environment) were paired with an electric shock (or “bee sting”) to the hand; flowers picked in the safe zone were never paired with a shock. Participants also collected and returned neutral objects as a measure of spatial memory. Galvanic skin response (GSR) was measured throughout the task and anxiety was assessed via the State Trait Anxiety Inventory (STAI). Participants were categorized as learners if they correctly identified the two zones after the task. Non-learners, compared to learners, performed significantly worse during the spatial memory task and demonstrated significantly higher state anxiety scores and GSR levels throughout the task. Learners showed higher skin conductance response (SCR) in the dangerous zone compared to the safe zone while non-learners showed no SCR differences between zones. These results indicate that state anxiety may impair spatial mapping, disrupting contextual threat learning.

## Introduction

Using spatial mapping processes to identify safe and dangerous areas in an environment is crucial for survival. Therefore, elucidating factors that affect the processes of spatial learning and navigation is essential to understanding why some individuals fail to discriminate between areas within their environment, leading to maladaptive behaviors. However, studies examining spatial mapping in threatening contexts have primarily focused on evaluating spatial memory performance under a threat of shock unrelated to the task (Bublatzky et al., 2023; Goodman et al., 2020), or only focused on those who successfully conditioned to a threat (Andreatta & Pauli, 2021; Suarez-Jimenez et al., 2018, 2021). Therefore, factors that affect one’s ability to learn about threats in an environment through spatial mapping processes are poorly understood, especially in individuals who do not learn to discriminate between contextual cues.

Traditionally, studies examining threat learning have used classical conditioning paradigms, where a neutral stimulus is paired with an aversive stimulus (e.g., electric shock). However, contextual threat conditioning links the aversive stimulus to an environmental context (e.g., presentation of a light; for a review, see Lonsdorf et al., 2017). Contextual conditioning can engage spatial mapping processes by directing attention to landmarks and forming a cognitive representation of the environment (Schwabe et al., 2007, 2010, 2012). Creation and utilization of this mental representation are considered crucial for successful conditioning and discrimination (Maren et al., 2013).

Research presents mixed findings regarding anxiety’s role in threat conditioning learning and discrimination. Some studies show no association between state (transitory emotional response; Joos et al., 2012) or trait anxiety (anxiety as an aspect of personality; Baas, 2013; Haaker et al., 2015; Torrents-Rodas et al., 2013) and learning. Other studies show individuals with high trait anxiety have heightened fear responses to conditioned safety or neutral cues (Cohen Kadosh et al., 2015; Gazendam et al., 2013; Haaker et al., 2015; Stegmann et al., 2019) and reduced threat and safety contingency awareness (Baas et al., 2008; Chan & Lovibond, 1996; Grillon, 2002). However, the impact of anxiety on contextual discrimination learning, especially focusing on those who do not learn the contingencies, has not been studied.

Few threat learning and discrimination studies have explored differences between those who acquire conditioning (i.e., learn to discriminate between threat and safety based on contextual cues) and those who do not (Baas, 2013; Baas et al., 2008; Chan & Lovibond, 1996; Kindt & Soeter, 2014). Two studies examined factors, such as anxiety and attention control, affecting threat learning based on both discrete and contextual cues (Baas, 2013; Baas et al., 2008). Trait anxiety was not significantly associated with contingency learning (Baas, 2013; Baas et al., 2008), but self-reported attentional control was significantly higher in those who learned cue contingencies compared to those who did not (Baas, 2013). In both studies, participants who did not learn both cue and context contingencies were excluded, and participants could not freely navigate or make any navigation-related decisions. In the current study, participants freely navigated a virtual environment where contingencies were based solely on contextual cues, allowing us to assess participants’ contextual threat learning and spatial mapping more accurately.

We previously described the behavioral and physiological profile of individuals who learned to discriminate between safety and threat within an environment (Suarez-Jimenez et al., 2018, 2021); however, results focused solely on individuals who learned the contingencies of the paradigm and did not explore how individual differences, such as state or trait anxiety, may affect learning. To our knowledge, no study has explored how individual differences affect acquisition during spatial threat learning. We postulate anxiety affects spatial mapping, hindering contextual threat learning. To understand how individual differences affect the processes of spatial mapping and threat discrimination, we compared the profiles of those who learned the contingencies of our VR contextual threat conditioning task (i.e., learners) with the profiles of those who did not learn (i.e., non-learners).

## Methods

### Participants.

A new sample of 70 healthy student volunteers (46 female) between 20 and 30 years old (mean age=22.99; SD=2.85) were recruited from the University College London (UCL). Participants were categorized as learners (n=50) if they correctly identified the safe and dangerous zones of the environment; participants were categorized as non-learners if they could not identify the zones. All volunteers were right-handed, free of neurological or psychological impairments, as indicated via self-report, and provided written informed consent before participating. After completing the study, participants were debriefed and reimbursed for their time. The UCL Research Ethics Committee approved this study.

### Pre-task questionnaire.

On the day of the study, prior to the task, participants completed the Spielberger State-Trait Anxiety Inventory (STAI) Forms Y-1 and Y-2 to assess state and trait anxiety, respectively, and the Raven Progressive Matrices to measure visual intelligence.

### VR Conditioning task.

**Figure 1.**
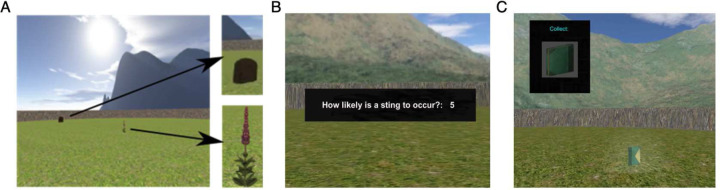
Task illustrations. A. The VR conditioning task, showing a beehive and a flower; B. Example of the scale participants used to rate their expectation of receiving a shock during the conditioning task; C. Example of one of the four objects participants collected and returned during the spatial memory task.

Participants were instructed to navigate a circular virtual environment and pick flowers that appeared one at a time in random locations (Figure 1A). Participants were informed that the flowers, which were identical in appearance, might contain a bee that could sting them, represented by a shock. Upon picking a flower, participants were prompted to rate their expectancy of receiving a shock on a scale from 0–9 (0 for no shock, 9 for definite shock; Figure 1B). After entering their rating, the participant’s avatar was held stationary for a period of 2000ms–8000ms, after which they could continue to move in the environment and another flower would appear. The task included 80 flowers. Half of the environment was associated with danger, where approximately 40 flowers were paired with shocks in 35% (n=12), 50% (n=29), or 60% (n=29) of trials; the other half of the environment was associated with safety, with the remaining 40 flowers never paired with shocks. While the experiments used three different reinforcement rates, overall, we found the same effects and patterns, albeit at different power, across the three reinforcement rates. Therefore, we pooled the data across the three reinforcement rate studies to increase the power of our analysis, and used the reinforcement rate as a factor of interest in the analysis. The full data analysis specific to each reinforcement rate can be found in the supplementary analysis, particularly for any significant interaction or main effect.

### Electrical stimulation.

A Digitimer DS7A electrical stimulator (Digitimer, Welwyn Garden City, UK) was used to deliver electrical stimulations (shocks) to the left hand through an Ag/AgCl electrode. Before the task, participants received a series of shocks, starting at 1.2 mA, and rated the level of pain of each shock on a scale of 1–10. Shock intensity was adjusted for each participant to a level that was described as uncomfortable but not painful.

### Galvanic skin response (GSR).

GSR was measured throughout the task as an index of anxiety via 8-mm Ag/AgCl electrodes attached to the middle phalanges of the index and middle fingers of the participant’s left hand. Data were acquired using a custom-built constant voltage coupler (2.5 V) with output converted into an optical pulse frequency. The optical signal was then converted to voltage pulses and recorded throughout the experiment (Micro 1401/Spike 2; Cambridge Electronic Design).

### Spatial memory task.

After every four trials of the conditioning task, participants performed a trial of a spatial memory task that required them to learn the locations of four different, non-valenced objects (wooden box, gas can, book, and clock; Figure 1C) that were never paired with shocks. Participants were asked to collect and later return the objects to their original locations. One object was located in each quadrant of the environment. After the participant returned an object to where they thought it was originally located, the object reappeared in its actual original location, regardless of where it was returned. Participants then had to collect the object again as a reminder of the original location for the next time they were asked to return it. Participants continued to collect and return objects for 16 trials (four iterations per object).

### Post-task questionnaire.

At the end of the task, participants were asked to identify the dangerous and safe zones of the environment, name the four objects, and describe the location of each object. Participants who correctly identified the dangerous and safe zones of the environment were categorized as learners; participants who could not were categorized as non-learners. After the task, all participants completed STAI Form Y-1 to measure state anxiety again.

## Data Analysis

### Spatial memory analysis.

Spatial memory of the virtual environment was measured by object placement distance error (i.e., the difference in distance from the location where the participant placed the object compared to the original location of the object). Object distance data were analyzed in R using repeated measures multivariate ANOVAs to test differences between groups (i.e., learners and non-learners), quarter (or iteration in the object distance), zone (i.e., danger and safety), and shock reinforcement rate (35%, 50%, and 60%). Tukey’s Honest Significant Differences (HSD) test was conducted post-hoc on significant findings to assess the directionality of the results.

Participants’ memory of the virtual environment was analyzed in R by averaging participants’ answers for the number of object names recalled and for the number of object locations recalled. A Welch two sample *t*-test was conducted for each measure to test differences between groups.

### STAI analysis.

Trait anxiety scores (as measured by STAI Trait Y-2) were analyzed in R using a multivariate ANOVA comparing groups and shock reinforcement rate. State anxiety scores (as measured by STAI State) were analyzed in R using a multivariate ANOVA comparing groups (learners and non-learners), time (pre-task and post-task Y-1), and shock reinforcement rate (35%, 50%, and 60%). Tukey’s HSD test was conducted post-hoc on significant findings to assess the directionality of the results.

### GSR analysis.

Data processing and analysis of skin conductance were performed using MATLAB. GSR data were down-sampled to 200 Hz and then synchronized to the task. GSR was assessed during two periods of the conditioning task. First, mean skin conductance level (SCL) during each trial was quantified as “tonic skin conductance level” as participants approached the flower. SCL was quantified based on the final three-quarters of the approach period, from flower appearance until the participant picked the flower. SCL was calculated by measuring the mean skin conductance from the beginning of the active approach until right before the participant picked the flower, for each trial. Second, skin conductance responses (SCR) were analyzed during the stationary period to examine phasic changes in anticipation of the shock outcome. SCRs were calculated for every trial by subtracting the minimum SCR during the stationary period from the maximum SCR (peak). Any response difference under 0.03 micro-Siemens was scored as zero. SCRs were log-transformed (log [1+SCR]) to normalize the distribution, and then range-corrected ([SCR-SCRmin]/[SCRmax-SCRmin]) to control for individual variation in response. The same correction was applied to the SCLs. For analyses, SCL and SCR data were averaged into equal quarters (quarter; Q1-Q4) across the duration of the experiment, with 10 trials in each quarter per condition (zone; danger, safety).

### Shock expectancy ratings analysis.

Each rating (0–9) was averaged across trials for each quarter (quarter; Q1-Q4) of the experiment per condition (zone; danger, safety), creating equal quarters of 10 trials for each condition.

GSR data and expectancy ratings were analyzed in R using repeated measures multivariate ANOVAs to test differences between groups (i.e., learners or non-learners), quarter (or iteration in the object distance), zone, and shock reinforcement rate. Tukey’s HSD test was conducted post-hoc on significant findings to assess the directionality of the results.

### Covariate analysis.

We used a Chi-square test in R to assess differences in sex and an independent-sample t-test in R to assess group differences in age and visual intelligence as per the Raven’s Progressive Matrices. We found no significant difference between the two groups in sex (*p*=.1888), age (*p*=.225), or visual intelligence (*p*=.461). Therefore, these were not added as covariates in the analysis.

We used an alpha value of 0.05 for all statistical tests, two-tailed, with 95%CI. All methods and analysis were performed following our previous studies using this paradigm (Suarez-Jimenez et al. 2018, 2021); see the papers for more details.

### Data accessibility.

Requests for data should be addressed to the corresponding author. The experiment was not pre-registered.

## Results

### Object placement distance error

**Figure 2.**
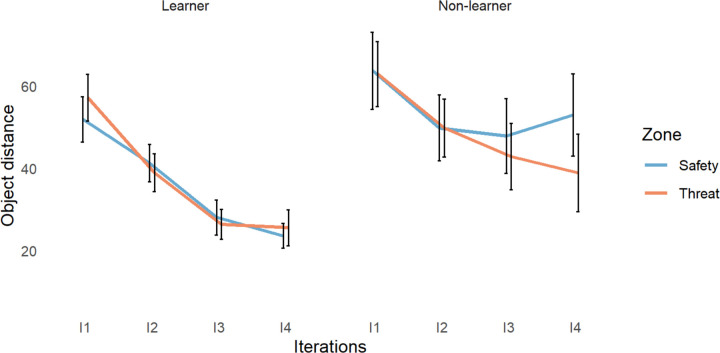
Changes in object distance error across iterations (I1–4) for learners and non-learners in the safe and dangerous zones of the environment.

We compared participants’ object placement to the original location of each object (distance error; Figure 2) using a 2×4×2×3 ANOVA with group (learner, non-learner), iteration (I1-I4), zone (danger, safety), and reinforcement rate (35%, 50%, 60%) as factors.

We found significant group by zone by reinforcement rate (*F*(2, 512)=3.13, *p*=.045), and group by reinforcement rate (*F*(2, 512)=13.02, *p*=3×10^−6^) interactions. We also found significant main effects of group (*F*(1, 512)=21.12, *p*=5×10^−6^) and iteration (*F*(3, 512)=18.09, *p*=4×10^−11^). All other results had a p-value above 0.1. For a full breakdown of the reinforcement rate effects, see the supplementary analysis (Supplementary figures 1 to 3).

To clarify the directionality of the main effects, we conducted Tukey’s HSD post hoc analysis. We found that learners, compared to non-learners, placed objects significantly closer to their original location (*p*=5×10^−6^, 95%CI[−21.762, −7.282]). Across participants in both groups, object distance error was significantly smaller on the 2^nd^ (*p*=.003, 95%CI[−24.615, −3.802]), 3^rd^ (*p*=1×10^−8^, 95%CI[−35.076, −14.263]), and 4^th^ (*p*=1×10^−9^, 95%CI[−36.812, −15.999]) iterations compared to the 1^st^ iteration, and significantly smaller on the 3^rd^ (*p*=.048, 95%CI[−20.868, −.055]) and 4^th^ (*p*=.014, 95%CI[−22.603, −1.790]) iterations compared to the 2^nd^ iteration.

### Object and location memory

We compared participants’ memory of the virtual environment, including the number of object names and locations they remembered, using a Welch Two Sample t-test.

We found no significant difference in the number of recalled object names between groups (*t*(19)=1.00, *p=*.330, 95%CI[−.055, .155]). However, we found a trending difference in the number of recalled object locations between groups (*t*(20.06)=1.82, *p*=.085, 95%CI[−.073, 1.053]), where learners, compared to non-learners, identified more object locations when asked at the end of the task.

### State-trait anxiety inventory (STAI)

We compared participants’ trait anxiety scores using a 2×3 ANOVA with group and reinforcement rate as factors. However, we found no significant differences in trait anxiety scores.

**Figure 3.**
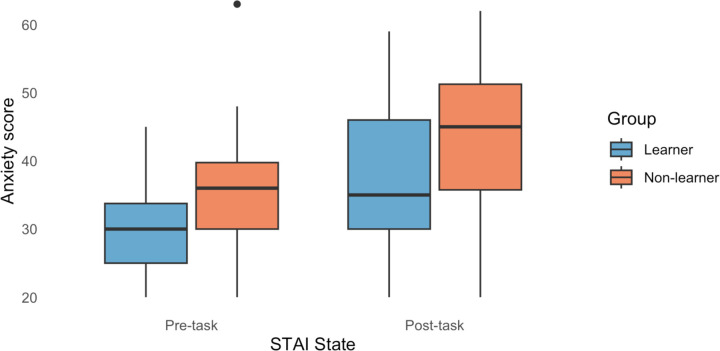
State Anxiety scores (STAI State) for learners and non-learners before and after the task. Learners had lower state anxiety scores than non-learners. Anxiety scores increased from pre- to post-task for all. Outliers are represented by a black dot ●.

Next, we compared participants’ state anxiety scores (Figure 3) using a 2×2×3 ANOVA with time (pre-task and post-task), group, and reinforcement rate as factors. We found a significant main effect of group (F(1)=14.31, *p*=2×10^−4^) and time (F(1)=21.98, p=6×10^−6^) in the state anxiety scores. All other results had a p-value above 0.1.

To clarify the directionality of the main effects, we conducted Tukey’s HSD post hoc analysis. We found that non-learners, compared to learners, had significantly higher combined state anxiety scores (*p*=2×10^−4^, 95%CI[3.165, 10.105]). Across both groups, pre-task state anxiety scores were significantly lower than post-task state anxiety scores (*p*=6×10^−6^, 95%CI[−10.564, −4.293]).

### SCL

**Figure 4.**
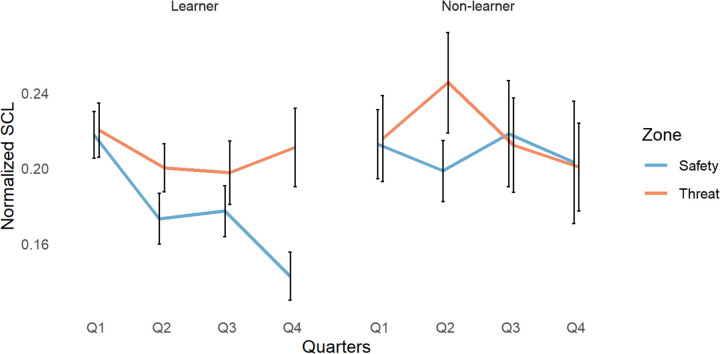
Changes in SCL across quarters (Q1–4) for learners and non-learners in safe and dangerous zones of the environment.

We compared participants’ skin conductance level (SCL; Figure 4) as they approached flowers, using a 2×4×2×3 ANOVA with group, quarter (Q1–4), zone, and reinforcement rate as factors.

We found significant group by reinforcement rate (*F*(2, 459)=6.43, *p*=.002) and quarter by reinforcement rate interactions (*F*(6,459)=2.75, *p*=.012). We also found a significant main effect of group (*F*(1, 459)=5.17, p=.023), quarter (*F*(3, 459)=3.02, *p*=.029), and zone (*F*(1, 459)=6.46, *p*=.011). All other results had a p-value above 0.1. For a full breakdown of the reinforcement rate effects, see the supplementary analysis (Supplementary figures 4 to 6).

To clarify the directionality of the main effects, we conducted Tukey’s HSD post hoc analysis. We found that learners, compared to non-learners, had significantly lower SCL across the task (*p*=.027, 95% C.I=[−.041, −.003]). Overall, participants in both groups had significantly higher SCL in the 1^st^ quarter compared with the 4^th^ quarter (*p*=.020, 95% C.I=[.004, .071]). Participants in both groups had significantly higher SCL in the dangerous zone of the environment compared with the safe zone (*p*=.013, 95% C.I=[.005, .039]).

### SCR

**Figure 5.**
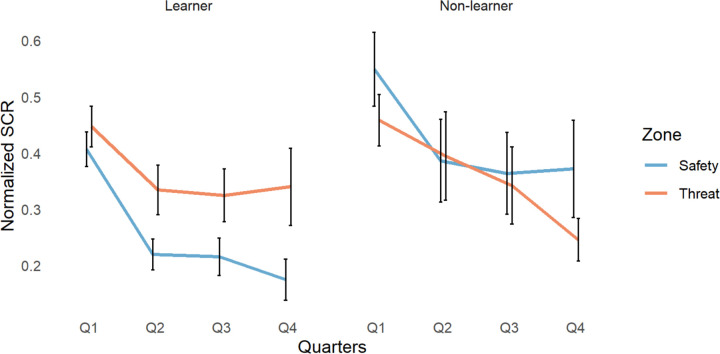
Changes in SCR across quarters (Q1–4) for learners and non-learners in safe and dangerous zones of the environment.

We compared skin conductance response (SCR; Figure 5), as participants were held stationary, using a 2×4×2×3 ANOVA with group, quarter, zone, and reinforcement rate as factors.

We found significant group by zone (*F*(1, 459)=7.91, *p*=.005) and quarter by reinforcement rate interactions (*F*(6,459)=2.622, *p*=.016). We also found a significant main effect of group (*F*(1, 459)=11.22, *p*=9×10^−4^), quarter (*F*(3, 459)=12.55, *p*=6×10^−8^), zone (*F*(1, 459)=6.05, *p*=.014), and reinforcement rate (*F*(2, 459)=21.90, *p*=8×10^−10^). All other results had a p-value above 0.1. For a full breakdown of the reinforcement rate effects, see the supplementary analysis (Supplementary figures 7 to 9).

To clarify the directionality of the findings, we conducted Tukey’s “Honest Significant Differences” post hoc analysis. We found that learners, compared to non-learners, had significantly lower SCR in the safe zone of the environment (*p*= 1×10^−4^, 95% C.I=[−.064, −.253]). Learners also had a significantly lower SCR in the safe zone of the environment compared with the dangerous zone (*p*=.002, 95% C.I=[−.027, −.171]). Overall, learners, compared to non-learners, had significantly lower SCR across the task (*p*=.001, 95% C.I=[−.034, −.140]). Participants in both groups had significantly higher SCR in the 1^st^ quarter compared to the 2^nd^ quarter (*p*=8×10^−5^, 95% C.I=[.221, .058]), 3^rd^ quarter (*p*=8×10^−6^, 95% C.I=[.238, .074]), and 4^th^ quarter (*p*=1×10^−6^, 95% C.I=[.280, .095]). Participants in both groups had significantly higher SCR in the dangerous zone of the environment compared to the safe zone (*p*=.014, 95% C.I=[.012, .104]).

### Expectancy ratings

**Figure 6.**
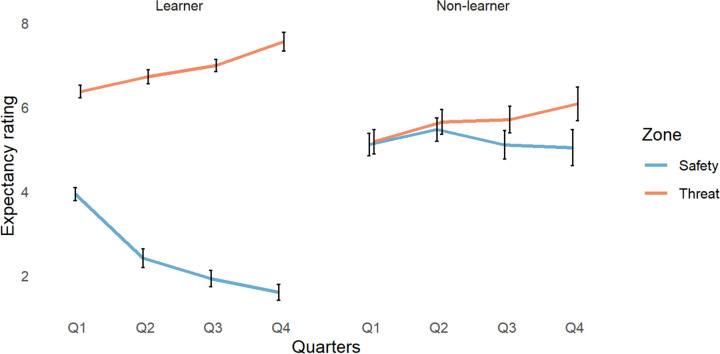
Changes in expectancy rating across quarters (Q1–4) for learners and non-learners in the safe and dangerous zones of the environment.

We compared participants’ shock expectancy ratings (Figure 6) using a 2×4×2×3 ANOVA with group, quarter, zone, and reinforcement rate as factors.

We found significant interactions in: group by quarter by zone (*F*(3, 439)=4.49, *p*=.004), group by quarter by reinforcement rate (*F*(1,439)=3.70, *p*=.026), group by zone (*F*(1, 439)=247.79, p=1×10^−16^), group by quarter (*F*(3,439)=4.91, *p*=.002), and quarter by zone (*F*(3, 439)=25.57, *p*=3×10^−15^). We found significant main effects of group (*F*(1, 439)=42.51, *p*=2×10^−10^), quarter (*F*(3, 439)=5.11, *p*=.002), zone (*F*(1, 439)=791.46, *p*=1×10^−16^), and reinforcement rate (*F*(2, 439)=5.52, *p*=.004). All other results had a p value above 0.1. For a full breakdown of the reinforcement rate effects, see the supplementary analysis (Supplementary figures 10 to 12).

To clarify the directionality of the findings, we conducted Tukey’s “Honest Significant Differences” post hoc analysis. We found that learners rated their expectancy of a shock significantly higher in the dangerous zone compared to the safe zone across all quarters (quarter 1 threat vs. safety: *p*=1×10^−16^, 95%CI[1.578, 3.330], quarter 2 threat vs. safety: *p*=1×10^−16^, 95%CI[3.457, 5.209], quarter 3 threat vs. safety: *p*=1×10^−16^, 95%CI[4.196, 5.957], and quarter 4 threat vs. safety: *p*=1×10^−16^, 95%CI[4.735, 6.920]). In the safe zone, learners gave significantly lower shock expectancy ratings in quarters 2 (*p* =5×10^−7^, 95%CI[−2.402, −.650]), 3 (*p*=1×10^−16^, 95%CI[−2.883 −1.131]) and 4 (*p*=1×10^−16^, 95%CI[−3.213, −1.332]) compared with quarter 1. Learners, compared to non-learners, rated their expectancy of shock significantly higher in the dangerous zone (*p*=1×10^−10^, 95%CI[1.639, .742]) and significantly lower in the safe zone (*p*=1×10^−16^, 95%CI[−2.204, −3.093]). Non-learners did not show a significant difference in shock expectancy ratings in the dangerous zone compared to the safe zone in any quarter of the experiment, and their ratings did not differ between quarters in either zone.

## Discussion

We found that non-learners displayed poorer spatial memory of their environment as measured by increased object placement distance error when placing the objects in the environment. Non-learners overgeneralized the presence of threat, as shown by their significantly higher SCR and threat expectancy ratings compared to learners in the safe zone. Non-learners, compared to learners, were more anxious overall, as measured by GSR and by state anxiety scores.

### Memory task performance

We found no significant differences between learners and non-learners when comparing recollection of the names of neutral objects (objects not associated with a threat) during the post-task interviews. However, learners, compared to non-learners, were significantly more accurate in recalling the location of neutral objects during the task, as measured by object placement distance error and number of object locations recalled during post-task interviews. These findings are consistent with previous research on emotional arousal and impaired spatial memory retrieval (Goodman et al., 2020; Schwabe et al., 2007), where anxiety was found to impair performance in a virtual spatial radial maze task and was associated with reduced use of spatial learning strategies. Additionally, non-learners did not learn the location of the danger zone, further suggesting impaired spatial mapping. Research in cognitive resources availability and management shows that severe, but not mild, induced anxiety negatively impacts participants’ cognitive task performance, suggesting that at some point the management of anxiety takes over the cognitive resources implicated in decision-making (Robinson et al., 2013). The electric shock used in this study was individually configured to be a mild stress inducer; however, it appears that higher anxiety during the task (as per GSR) overwhelmed non-learners during the spatial learning of valenced objects. Another potential explanation is that anxiety triggers compensatory strategies like enhanced attention allocation toward potential threats (Robinson et al., 2013; Sandi, 2013), which may bias attention away from the environmental context that is actually predictive of where threat is located.

### State vs trait anxiety

Non-learners, compared to learners, had significantly higher state anxiety scores. Interestingly, we did not find any differences in trait anxiety between groups. Transient arousal (state anxiety) and the presence of threat may influence spatial mapping in non-learners via attention allocation. According to attentional control theory, anxiety may impair performance via increased competition among attentional resources, thereby reducing attention to the ongoing task (Eysenck et al., 2007). Indeed, state and trait anxiety have been linked to poor attentional control and reduced cognitive performance in tasks where task irrelevant threat-related or neutral stimuli are present (Berggren & Derakshan, 2013; Eysenck et al., 2007; Pacheco-Unguetti et al., 2010). One study claims that although both state and trait anxiety are related to attentional control deficits, therefore affecting cognitive performance, the two may affect different networks of attention in distinct ways (Pacheco-Unguetti et al., 2010). Additionally, in uncertain situations, anxiety allocates attentional resources to threat detection mechanisms (Bishop, 2008) and is associated with attentional bias toward threat-related stimuli (Bar-Haim et al., 2007; Berggren & Derakshan, 2013; Eysenck et al., 2007). Non-learners of our task may have an increased attention toward threat-related stimuli, which could be attributed to a feeling of uncertainty during the task, and therefore their more anxious state. Failure to inhibit this attentional bias may result in a lack of attention to the surrounding environment, which contains contextual cues (e.g., local and distal landmarks) that are useful for the creation of a cognitive map and the prediction of the shock contingencies. Thus, non-learners may not have a well-defined mental representation of the environment - hindering their ability to discriminate between safe and dangerous zones of the environment.

### GSR and overgeneralization of threat

Non-learners, compared to learners, showed significantly higher SCR and threat expectancy ratings in the safe zone. In other words, while learners showed differential responses to the safe and dangerous zones, suggesting successful discrimination, non-learners did not. Instead, non-learners displayed hyperarousal in the safe zone, suggesting that non-learners overgeneralized the presence of threat. Conditioning studies also show that healthy adults can discriminate between safe and threatening cues (Duits et al., 2015; Lissek et al., 2005), and our results support these findings in the learner group. In the non-learner group, decreased performance on the spatial memory task may provide insight as to why they were unable to discriminate between safe and dangerous zones of the environment. Non-learners, who were unable to accurately locate neutral objects in the environment, could lack an accurate mental representation of the environment itself. Lack of an accurate mental representation of the environment could hinder discrimination learning, especially when location in the environment is predictive of the aversive stimulus. Furthermore, in an environment where threat occurs based on location, having a poor cognitive map of threat in the environment could induce additional anxiety, as it becomes uncertain to the participant when they will receive a shock. This uncertainty could induce anxiety during the task and explain physiological hyperarousal and increased expectation of threat in safe zones. Previous work has shown that healthy adults with task-induced anxiety show disruption in conditioning acquisition (Robinson et al., 2013). Moreover, conditioning studies in anxiety disorders and posttraumatic stress disorders (PTSD) have shown similar disruptions in conditioning acquisition (Lissek et al., 2005, 2010). Specifically, participants with pathological anxiety and healthy participants with task-induced anxiety show overall higher arousal in response to safe cues (Duits et al., 2015; Lissek et al., 2010; Robinson et al., 2013). Likewise, non-learners in this study overgeneralized the threat, responding with higher arousal toward cues that resembled threatening cues, even in the safe zone. While past research primarily employed models of conditioning that used stationary images or video, this study used free exploration of a naturalistic VR environment, expanding the knowledge about the effect of anxiety on spatial mapping and navigation. It is important to note that in a recent study (Suarez-Jimenez et al., 2021), we found that patients with generalized anxiety disorder (GAD) still learned the contingencies of the task, although they engaged different brain networks to complete the task than those engaged by healthy participants. However, this study only focused on learners, and did not include non-learners in either group. Furthermore, this study showed similar SCR, SCL, and expectancy rating patterns in both healthy adult learners and adult learners with GAD, suggesting that the mental state (anxiety state) during the task might predict the ability to create an accurate mental representation of the environment, attend to the environmental cue, and therefore discriminate between safe and threatening areas. Nonetheless, it is possible that deficits in discrimination learning in non-learners of the current study cannot be solely explained by heightened anxiety during the task.

### Limitations

There are several limitations of this study that should be noted. First, the sample sizes (learners and non-learners) are unbalanced, and the size of the non-learner sample is small relative to the learner sample. To our knowledge, this is the first study to look at individual differences in learning during a location-based threat conditioning paradigm in which participants can freely navigate a virtual environment. Therefore, further studies should continue to explore these differences in bigger samples. Second, we pooled the data from three experiments that used different reinforcement rates to increase the power of statistical analysis. It is important to note that while the experiments used different reinforcement rates, we saw no significant differences between the experiments in the outcome measures of interest. Overall, we found the same effects and patterns, albeit at different power, across three reinforcement rates. While integrating the cohorts allowed us to see the differences in anxiety between learners and non-learners, we suggest viewing our findings as preliminary, and this work should be substantiated by further studies. Finally, our study did not focus on the mental health history of participants to assess trauma. All participants reported no past or present history of psychopathology but trauma was not assessed. Furthermore, the study did not assess other potential channels of learning that could explain the differences between groups, such as attention allocation and selection history. We recommend that future studies assess trauma and examine attention allocation during the formation of mental maps to further understand the underlying mechanisms of forming, updating, and maintaining an accurate mental representation of the environment.

## Conclusion

This is the first study to explore individual differences affecting spatial memory and contextual threat learning within a single, naturalistic VR environment that participants were able to freely explore. We found that impaired discrimination learning and impaired spatial memory are associated with greater anxiety when navigating an environment where threat is present. These findings contribute to the understanding of the effect of anxiety on navigational abilities and spatial learning. We suggest that future studies include an attention allocation assessment (e.g., eye-tracking) to clarify the mechanism behind some participants’ inability to learn safety cues, and a more detailed psychological assessment of participants that includes trauma exposure to rule out the possibility of any past or current undiagnosed psychological disorders.

## Supplementary Material

1 **Supplementary Figure 1.** Changes in object distance error across iterations (I1-I4) for learners and non-learners in the safe and dangerous zones of the environment at a 35% reinforcement rate.
**Supplementary Figure 2.** Changes in object distance error across iterations (I1–4) for learners and non-learners in the safe and dangerous zones of the environment at a 50% reinforcement rate.
**Supplementary Figure 3**. Changes in object distance error across iterations (I1–4) for learners and non-learners in the safe and dangerous zones of the environment at a 60% reinforcement rate.
**Supplementary Figure 4.** Changes in SCL across quarters (Q1–4) for learners and non-learners in safe and dangerous zones of the environment at a 35% reinforcement rate.
**Supplementary Figure 5.** Changes in SCL across quarters (Q1–4) for learners and non-learners in safe and dangerous zones of the environment at a 50% reinforcement rate.
**Supplementary Figure 6.** Changes in SCL across quarters (Q1–4) for learners and non-learners in safe and dangerous zones of the environment at a 60% reinforcement rate.
**Supplementary Figure 7.** Changes in SCR across quarters (Q1–4) for learners and non-learners in safe and dangerous zones of the environment at a 35% reinforcement rate.
**Supplementary Figure 8.** Changes in SCR across quarters (Q1–4) for learners and non-learners in safe and dangerous zones of the environment at a 50% reinforcement rate.
**Supplementary Figure 9.** Changes in SCR across quarters (Q1–4) for learners and non-learners in safe and dangerous zones of the environment at a 60% reinforcement rate.
**Supplementary Figure 10.** Changes in expectancy rating across quarters (Q1–4) for learners and non-learners in the safe and dangerous zones of the environment at a 35% reinforcement rate.
**Supplementary Figure 11.** Changes in expectancy rating across quarters (Q1–4) for learners and non-learners in the safe and dangerous zones of the environment at a 50% reinforcement rate.
**Supplementary Figure 12.** Changes in expectancy rating across quarters (Q1–4) for learners and non-learners in the safe and dangerous zones of the environment at a 60% reinforcement rate.
