# Supplementary material for: Using Virtual Reality to Study Spatial Mapping and Threat Learning
Source: Res Sq. 2024 Apr 3:rs.3.rs-3891586. Preprint. [Version 1] doi: 10.21203/rs.3.rs-3891586/v1 (PMC11030523; doi:10.21203/rs.3.rs-3891586/v1)
Supplement: 1 — Supplementary Figure 1. Changes in object distance error across iterations (I1-I4) for learners and non-learners in the safe and dangerous zones of the environment at a 35% reinforcement rate. Supplementary Figure 2. Changes in object distance error across iterations (I1–4) for learners and non-learners in the safe and dangerous zones of the environment at a 50% reinforcement rate. Supplementary Figure 3. Changes in object distance error across iterations (I1–4) for learners and non-learners in the safe and dangerous zones of the environment at a 60% reinforcement rate. Supplementary Figure 4. Changes in SCL across quarters (Q1–4) for learners and non-learners in safe and dangerous zones of the environment at a 35% reinforcement rate. Supplementary Figure 5. Changes in SCL across quarters (Q1–4) for learners and non-learners in safe and dangerous zones of the environment at a 50% reinforcement rate. Supplementary Figure 6. Changes in SCL across quarters (Q1–4) for learners and non-learners in safe and dangerous zones of the environment at a 60% reinforcement rate. Supplementary Figure 7. Changes in SCR across quarters (Q1–4) for learners and non-learners in safe and dangerous zones of the environment at a 35% reinforcement rate. Supplementary Figure 8. Changes in SCR across quarters (Q1–4) for learners and non-learners in safe and dangerous zones of the environment at a 50% reinforcement rate. Supplementary Figure 9. Changes in SCR across quarters (Q1–4) for learners and non-learners in safe and dangerous zones of the environment at a 60% reinforcement rate. Supplementary Figure 10. Changes in expectancy rating across quarters (Q1–4) for learners and non-learners in the safe and dangerous zones of the environment at a 35% reinforcement rate. Supplementary Figure 11. Changes in expectancy rating across quarters (Q1–4) for learners and non-learners in the safe and dangerous zones of the environment at a 50% reinforcement rate. Supplementary Figure 12. Changes in expec [file NIHPPrs3891586V1-supplement-1.pdf]

Supplementary Material

Results

Object placement distance error

To clarify the reinforcement rate interaction, we repeated 2x4x2x3 ANOVA with group (learner, non-learner), iteration (I1-I4), zone (safety, threat), and reinforcement rate (35%, 50%, 60%) as factors on each reinforcement rate separately. The group by zone interaction was significant only in 35% reinforcement rate ($F(1, 80)=5.38$, $p=.023$). The main effect of group was significant only in 35% ($F(1, 80)=27.62$, $p=1\text{x}10^{-6}$) and 60% ($F(1, 216)=10.60$, $p=.001$) reinforcement rates. The main effect of iteration was only significant in 50% ($F(3, 216)=9.65$, $p=5\text{x}10^{-6}$) and 60% ($F(3, 216)=7.90$, $p=5\text{x}10^{-5}$) reinforcement rates. All other results had a p value above 0.1.

To clarify the directionality of the main effects in 35% reinforcement rate (Supplementary Figure 1), we conducted Tukey's HSD post hoc analysis. We found that in the safe zone, learners, compared with non-learners, placed objects significantly closer to their original location ($p=5\text{x}10^{-6}$, 95%CI[-84.785, -29.026]). Overall, learners, compared with non-learners, placed objects significantly closer to their original location ($p=1\text{x}10^{-6}$, 95%CI[-54.437, -24.533]).

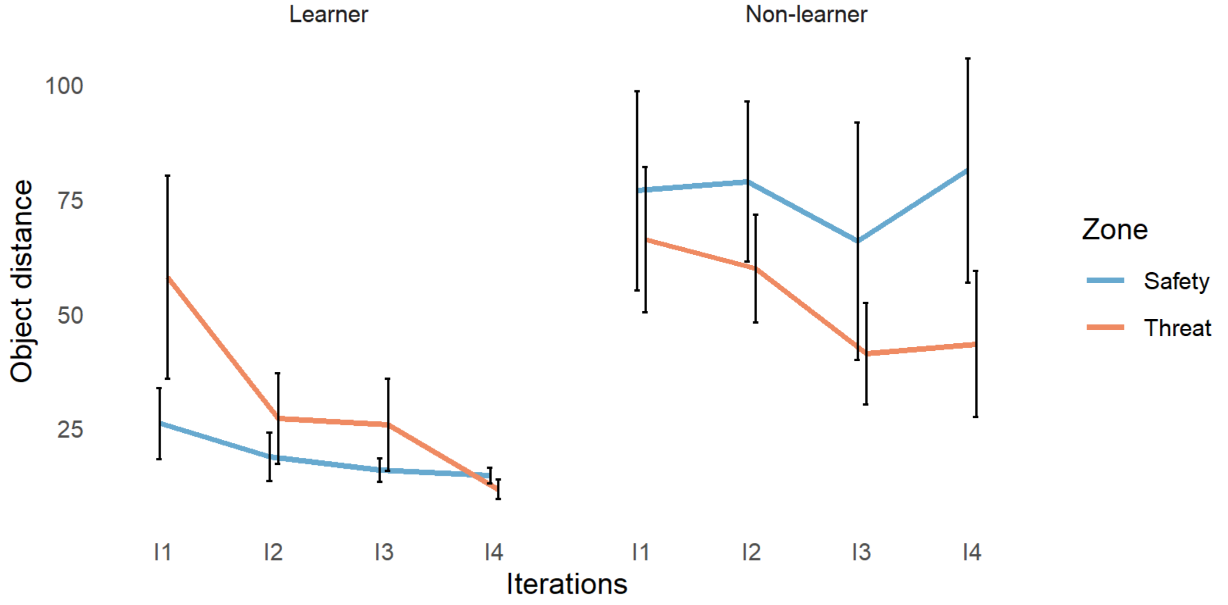

Supplementary Figure 1. Changes in object distance error across iterations (I1-I4) for learners and non-learners in the safe and dangerous zones of the environment at a 35% reinforcement rate.

To clarify the directionality of the main effects in 50% reinforcement rate (Supplementary Figure 2), we conducted Tukey's HSD post hoc analysis. We found that, compared to the 1st iteration, object distance error was significantly smaller in the 3rd and 4th iterations (p=1x10^{-4}, 95%CI[-38.989, -9.767] and p=2x10^{-5}, 95%CI[-41.646, -12.425] respectively). Compared to the 2nd iteration, object distance error was significantly smaller in the 4th iteration (p=.050, 95%CI[-29.225, -.003]).

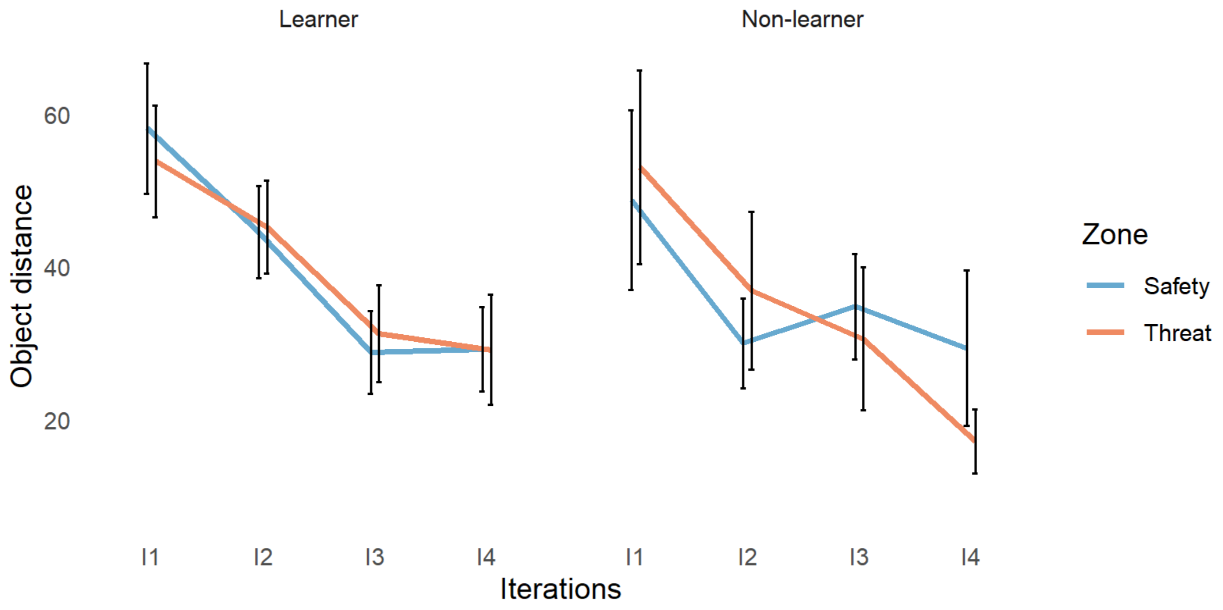

Supplementary Figure 2. Changes in object distance error across iterations (I1-4) for learners and non-learners in the safe and dangerous zones of the environment at a 50% reinforcement rate.

To clarify the directionality of the main effects in 60% reinforcement rate (Supplementary Figure 3), we conducted Tukey's HSD" post hoc analysis. We found that learners, compared with non-learners, placed objects significantly closer to their original location (p=.001, 95%CI[-27.464, -6.751]). We found that, compared to the 1st iteration, object distance error was significantly smaller in the 2nd, 3rd, and 4th iterations (p=.045, 95%CI[-34.679, -.283]; p=4x10^{-4}, 95%CI[-44.242, -9.846]; and p=1x10^{-4}, 95%CI[-46.016, -11.620] respectively).

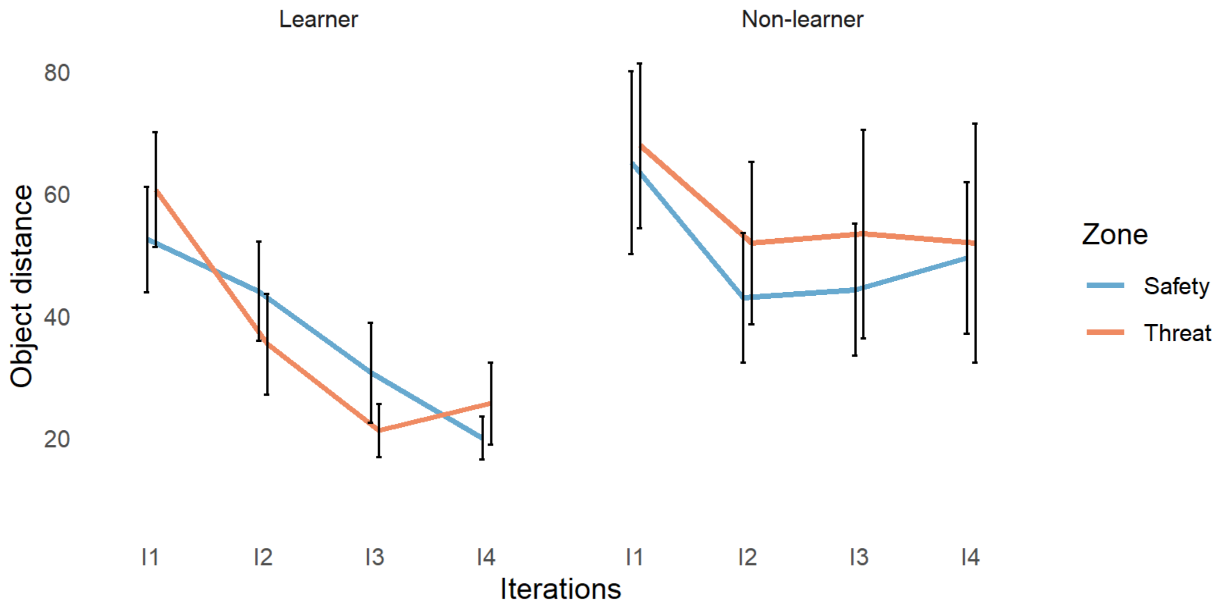

Supplementary Figure 3. Changes in object distance error across iterations (I1-4) for learners and non-learners in the safe and dangerous zones of the environment at a 60% reinforcement rate.

GSR and Expectancy Rating Results

SCL

To clarify the group by reinforcement rate interaction, we repeated 2x4x2x3 ANOVA with group, quarter, zone, and reinforcement rate as factors on each reinforcement rate separately. We found a significant main effect of group ($F(1, 188)=16.92$, $p=6\times10^{-5}$), quarter ($F(3, 188)=8.65$, $p=2\times10^{-5}$), and zone (F(1, 188)=4.78, $p=.030$) in 60% reinforcement rate. We found a trending main effect of zone ($F(1, 201)=2.80$, $p=.096$) in 50% reinforcement rate. All other results had a p value above 0.1.

To clarify the directionality of the main effects in 35% reinforcement rate (Supplementary Figure 4), we conducted Tukey's "Honest Significant Differences" post hoc analysis but none of the results of interest reached significance.

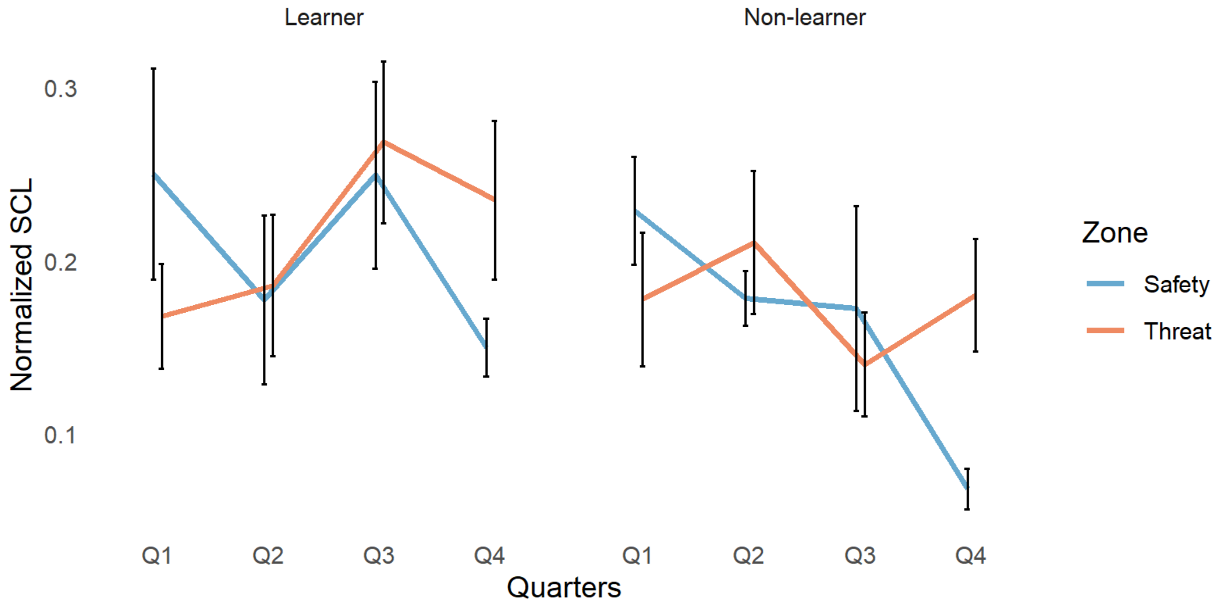

Supplementary Figure 4. Changes in SCL across quarters (Q1-4) for learners and non-learners in safe and dangerous zones of the environment at a 35% reinforcement rate.

To clarify the directionality of the main effects in 50% reinforcement rate (Supplementary Figure 5), we conducted Tukey's HSD post hoc analysis. We found that participants in both groups had a trending higher SCL in the dangerous zone of the environment compared with the safe zone (p=.096, 95%CI[-.004, .050]).

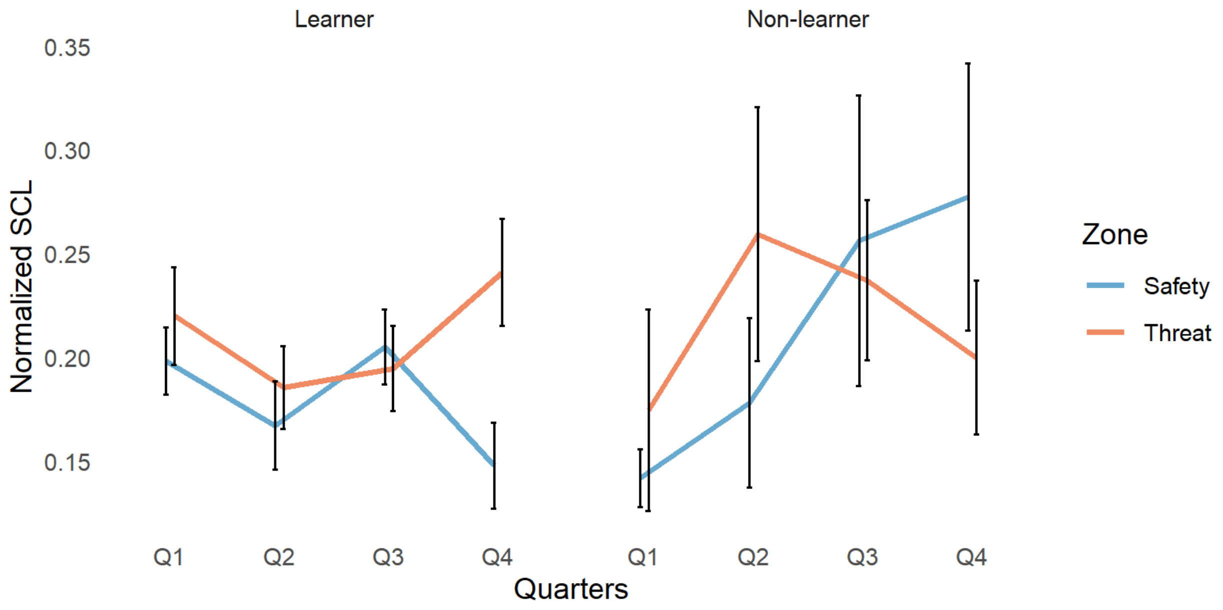

Supplementary Figure 5. Changes in SCL across quarters (Q1-4) for learners and non-learners in safe and dangerous zones of the environment at a 50% reinforcement rate.

To clarify the directionality of the main effects in 60% reinforcement rate (Supplementary Figure 6), we conducted Tukey's HSD post hoc analysis. We found that, overall, learners, compared to non-learners, had higher SCL (p=6x10^{-5}, 95%CI[.030, .086]). Compared to the 1st quarter, SCL was lower in the 3rd (p=.001, 95%CI[-.110, -.023]) and 4th quarters (p=1x10^{-4}, 95%CI[-.138, -.036]); compared to the 2nd quarter, SCL was lower in the 4th quarter (p=.020, 95%CI[-.108, -.007]). Participants in both groups had higher SCL in the dangerous zone of the environment compared with the safe zone (p=.032, 95%CI[.002, .052]).

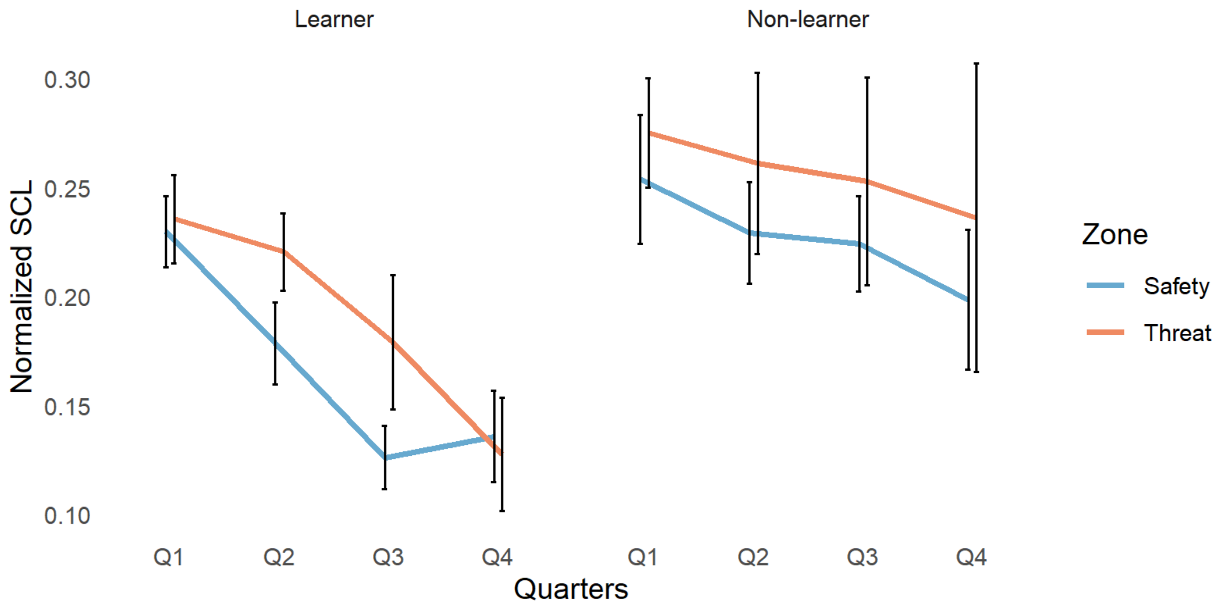

Supplementary Figure 6. Changes in SCL across quarters (Q1-4) for learners and non-learners in safe and dangerous zones of the environment at a 60% reinforcement rate.

SCR

To clarify the main effect of reinforcement rates, we repeated 2x4x2x3 ANOVA with group, quarter, zone, and reinforcement rate as factors on each reinforcement rate separately. We found a trending group by zone interaction in 35% ($F(1, 70)=2.99$, $p=.088$), 50% ($F(1, 201)=3.79$, $p=.053$), and 60% ($F(1, 188)=3.43$, $p=.066$) reinforcement rates. We found a significant main effect of group in 50% ($F(1,201)=8.06$, $p=.005$) and 60% ($F(1,188)=12.81$, $p=4x10^{-4}$) reinforcement rates and a trending effect of group in the 35% reinforcement rate($F(1,70)=3.75$, $p=.057$). We found a significant main effect of quarters in 35% ($F(1, 70)=3.09$, $p=.033$) and 60% ($F(1, 188)=50.56$, $p=1x10^{-16}$) reinforcement rates. All other results had a p value above 0.1.

To clarify the directionality of the main effects in 35% reinforcement rate (Supplementary Figure 7), we conducted Tukey's HSD post hoc analysis. We found that learners, compared with non-learners, had trending lower SCR in the safe zone (p=.065, 95%CI[-.277, .006]). Overall, learners, compared with non-learners, had trending lower SCR (p=.057, 95%CI[-.149, .002]). SCR was lower in the 4th quarter than the 1st quarter (p=.030, 95%CI[-.324, -.012]).

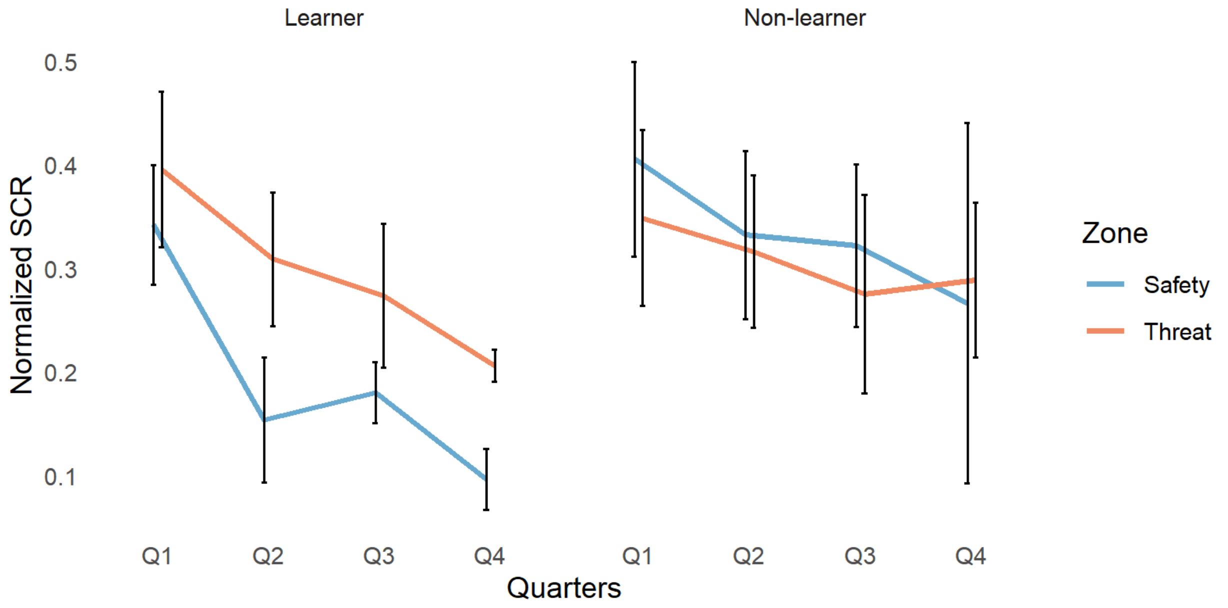

Supplementary Figure 7. Changes in SCR across quarters (Q1-4) for learners and non-learners in safe and dangerous zones of the environment at a 35% reinforcement rate.

To clarify the directionality of the main effects in 50% reinforcement rate (Supplementary Figure 8), we conducted Tukey's HSD" post hoc analysis. We found

that learners, compared with non-learners, had lower SCR in the safe zone (p=.005, 95%CI[-.510, -.067]). Overall, learners, compared with non-learners, had lower SCR (p=.005, 95%CI[-.288,-.052]). SCR was lower in the 4th quarter than the 1st quarter (p=.030, 95%CI[-.324, -.012]).

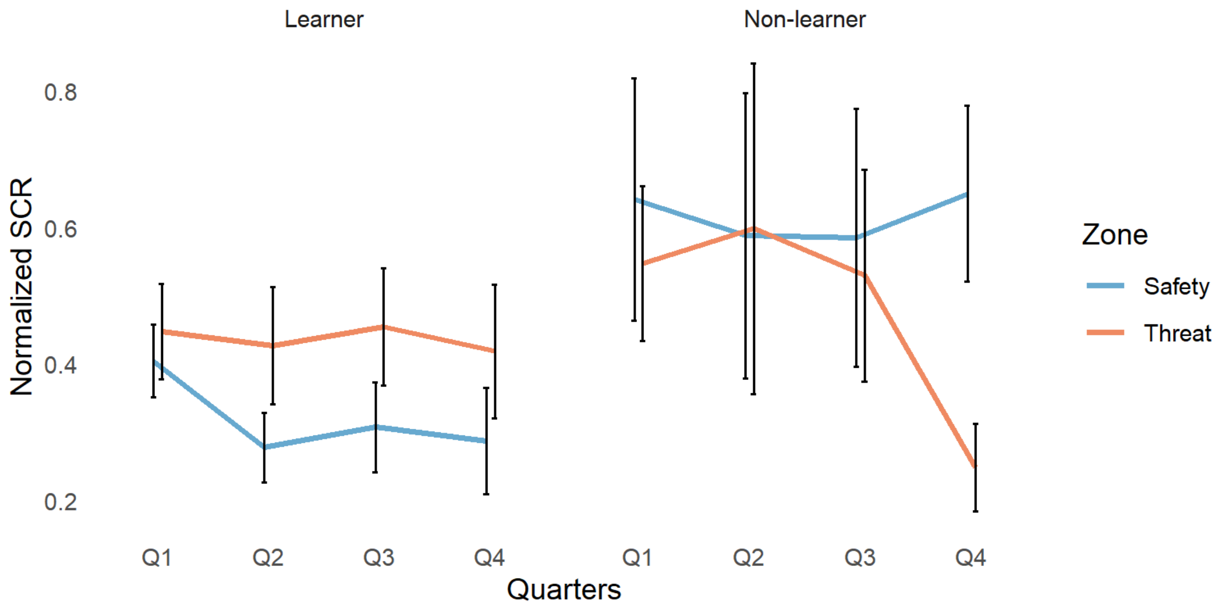

Supplementary Figure 8. Changes in SCR across quarters (Q1-4) for learners and non-learners in safe and dangerous zones of the environment at a 50% reinforcement rate.

To clarify the directionality of the main effects in 60% reinforcement rate (Supplementary Figure 9), we conducted Tukey's HSD" post hoc analysis. We found that, compared to the 1st quarter, learners had lower SCR in the 2nd ($p=3\times10^{-10}$, 95%CI[-.338, -.137]), 3rd ($p=1\times10^{-13}$, 95%CI[-.395, -.191]) and 4th ($p=2\times10^{-13}$, 95%CI[-.446, -.210]) quarters. Non-learners had lower SCR in the 4th quarter than the 1st quarter

(p=1x10^{-5}, 95%CI[-.521, -.136]). Overall, learners, compared with non-learners, had lower SCR (p=4x10^{-4}, 95%CI[-.131, -.038]). Compared to the 1st quarter, both groups had lower SCR in the 2nd (p=7x10^{-14}, 95%CI[-.311, -.167]), 3rd (p=4x10^{-14}, 95%CI[-.368, -.221]), and 4th (p=4x10^{-14}, 95%CI[-.413, -.243]) quarters, and, compared to the 2nd quarter, lower in the 4th (p=.036, 95%CI[-.174, -.004]) quarter.

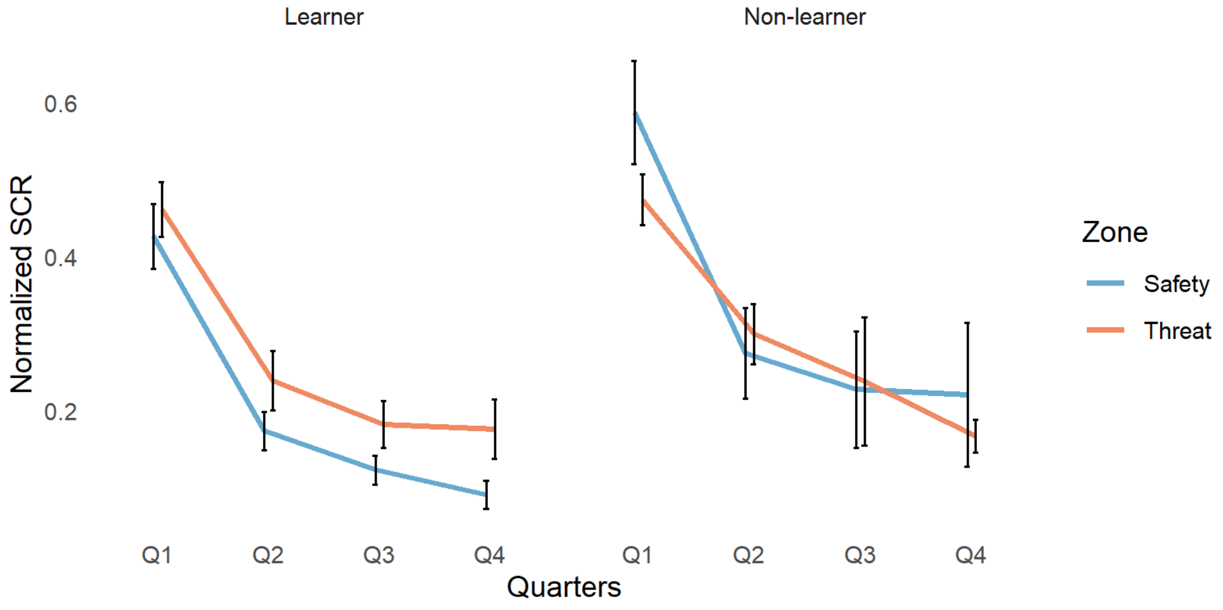

Supplementary Figure 9. Changes in SCR across quarters (Q1-4) for learners and non-learners in safe and dangerous zones of the environment at a 60% reinforcement rate.

Expectancy ratings

To clarify the effect of reinforcement rates, we repeated the 2x4x2x3 ANOVA with group, quarter, zone, and reinforcement rate as factors on each reinforcement rate

separately. The group by quarter by zone interaction was significant at a 35% reinforcement rate ($F(3, 70)=4.02$, $p=.011$). The quarter by zone interaction was significant across all reinforcement rates (35%: $F(3, 70)=5.03$, $p=.003$; 50%: $F(3, 201)=14.40$, $p=2\text{x}10^{-8}$; 60%: $F(3, 168)=6.84$, $p=2\text{x}10^{-4}$). The group by zone interaction was significant across all reinforcement rates (35%: $F(1, 70)=76.79$, $p=7\text{x}10^{-13}$; 50%: $F(1, 201)=113.49$, $p=1\text{x}10^{-16}$; 60%: $F(1, 168)=52.90$, $p=1\text{x}10^{-11}$). The group by quarter interaction was significant at a 50% reinforcement rate ($F(3, 201)=3.82$, $p=.011$) and trending at a 60% reinforcement rate ($F(3, 168)=2.53$, $p=.059$). The main effect of group was significant across all reinforcement rates (35%: $F(1, 70)=6.81$, $p=.011$; 50%: $F(1, 201)=15.70$, $p=1\text{x}10^{-4}$; 60%: $F(1, 168)=31.40$, $p=8\text{x}10^{-8}$). The main effect of quarter was significant at 35% ($F(3, 70)=5.33$, $p=.002$) and 60% ($F(3, 168)=5.64$, $p=.001$) reinforcement rates, and trending at 50% reinforcement rate ($F(3, 201)=2.16$, $p=.094$). The main effect of zone significant across all reinforcement rates (35%: $F(1, 70)=119.18$, $p=1\text{x}10^{-16}$; 50%: $F(1, 201)=445.49$, $p=1\text{x}10^{-16}$; 60%: $F(1, 168)=229.84$, $p=1\text{x}10^{-16}$). All other results had a p value above 0.1.

To clarify the directionality of the main effects in 35% reinforcement rate (Supplementary Figure 10), we conducted Tukey’s HSD” post hoc analysis. We found that , in the safe zone of the environment, learners rated the expectancy of the shock lower in the 3rd ($p=.006$, 95%CI[-4.678, -.422]) and 4th ($p=.001$, 95%CI[-5.188, -.725]) quarters than the 1st quarter. In the safe zone of the environment, in the 2nd quarter, learners rated the expectancy of the shock lower than non-learners ($p=.001$, 95%CI[-4.961, -.706]). Learners rated the expectancy of the shock higher in the dangerous

zone than in the safe zone in the 2nd (p=3x10^{-8}, 95%CI[2.289, 6.545]), 3rd (p=1x10^{-16}, 95%CI[3.672, 7.928]), and 4th (p=2x10^{-8}, 95%CI[3.356, 9.524]) quarters. In the safe zone, in the 3rd quarter, learners rated the expectancy of the shock lower than non-learners (p=2x10^{-4}, 95%CI[-5.261, -1.006]). In the dangerous zone, in the 3rd quarter, learners rated the expectancy of the shock higher than non-learners (p=.019, 95%CI[.289, 4.545]). In the safe zone, both groups rated the expectancy of the shock lower in the 3rd (p=.038, 95%CI[-2.690, -.044]) and 4th (p=2x10^{-4}, 95%CI[-3.905, -.823]) quarters than the 1st quarter, and the 4th quarter than the 2nd (p=.010, 95%CI[-3.355, -.273]) quarter. Both groups rated the expectancy of the shock higher in the dangerous zone than in the safe zone in the 2nd (p=1x10^{-3}, 95%CI[1.041, 3.687]), 3rd (p=1x10^{-5}, 95%CI[1.724, 4.370]), and 4th (p=2x10^{-8}, 95%CI[1.789, 5.253]) quarters. Learners rated the expectancy of the shock higher in threat than in safe zone (p=1x10^{-16}, 95%CI[3.553, 5.223]). In the safe zone, learners rated the expectancy of the shock lower than non-learners (p=1x10^{-9}, 95%CI[-3.188, -1.518]). In the dangerous zone, learners rate the expectancy of the shock higher than non-learners (p=5x10^{-5}, 95%CI[.693, 2.362]). Overall, ratings of the shock expectancy were higher in the dangerous zone than in the safe zone (p=1x10^{-16}, 95%CI[1.990, 2.882]). Overall, ratings of the shock expectancy were lower in the 4th quarter than the 1st (p=.004, 95%CI[-2.153, -.317]) and 2nd (p=.004, 95%CI[-2.153, -.317]) quarter. Overall, learners rated the shock expectancy lower than non-learners (p=.011, 95%CI[-1.030, -.138]).

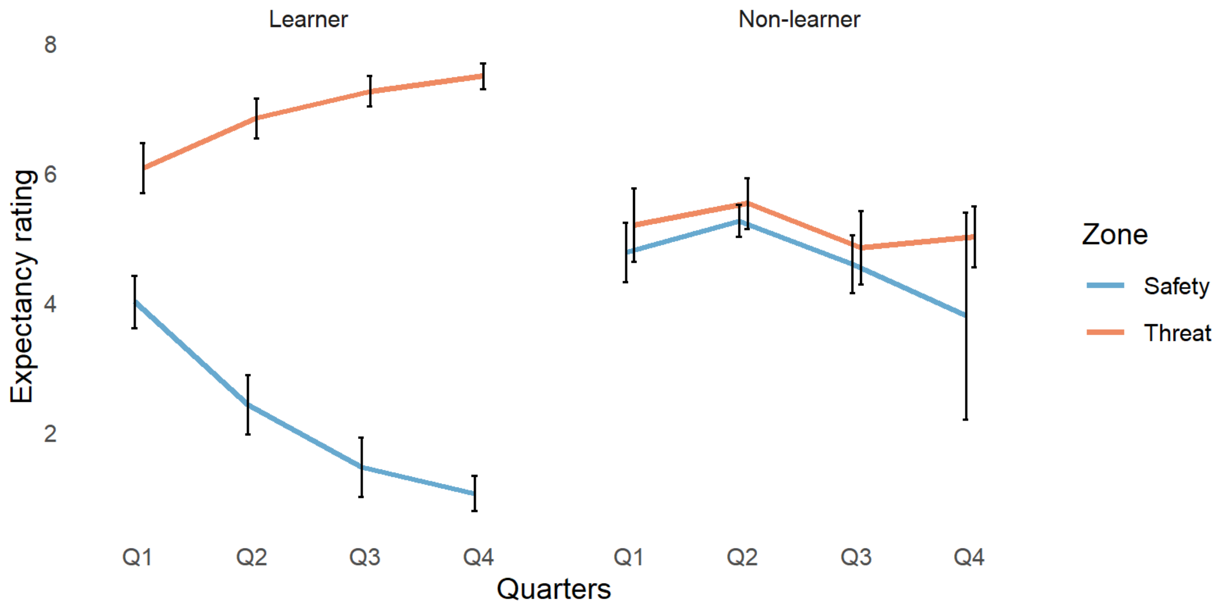

Supplementary Figure 10. Changes in expectancy rating across quarters (Q1-4) for learners and non-learners in the safe and dangerous zones of the environment at a 35% reinforcement rate.

To clarify the directionality of the main effects in 50% reinforcement rate (Supplementary Figure 11), we conducted Tukey's "Honest Significant Differences" post hoc analysis. We found that, in the safe zone of the environment, learners rated the expectancy of the shock lower in the 2nd (p=1x10^{-4}, 95%CI[-3.184, -.572]), 3rd (p=2x10^{-6}, 95%CI[-3.510, -.899]), and 4th (p=1x10^{-6}, 95%CI[-3.934, -1.051]) quarters compared to the 1st quarter. In the safe zone of the environment, learners rated the expectancy of the shock lower than non-learners in the 2nd (p=1x10^{-6}, 95%CI[-5.514,-1.454]), 3rd (p=7x10^{-8}, 95%CI[1.780, 5.840]), and 4th (p=2x10^{-7}, 95%CI[2.056, 7.007]) quarters. In the safe zone, learners rated the expectancy of the shock lower than non-learners (p=2x10^{-4}, 95%CI[-5.261, -1.006]) in the 3rd quarter. In the dangerous zone, learners rated the

expectancy of the shock higher than non-learners (p=.019, 95%CI[.289, 4.545]) in the 3rd quarter. Learners rated the expectancy of the shock higher in threat than in safe zone (p=1x10^{-16}, 95%CI[4.096, 5.104]). In the safe zone, learners rated the expectancy of the shock lower than non-learners (p=3x10^{-14}, 95%CI[-3.940,-2.358]). In the dangerous zone, learners rate the expectancy of the shock higher than non-learners (p=3x10^{-15}, 95%CI[-2.178, -.628]). In the 3rd quarter, learners rated the expectancy of the shock lower than non-learners (p=.022, 95%CI[-2.651,-.117]). Overall, ratings of the shock expectancy were higher in the dangerous zone than in the safe zone (p=1x10^{-16}, 95%CI[3.314, 3.998]). Overall, learners rated the shock expectancy lower than non-learners (p=1x10^{-4}, 95%CI[-1.268,-.425]).

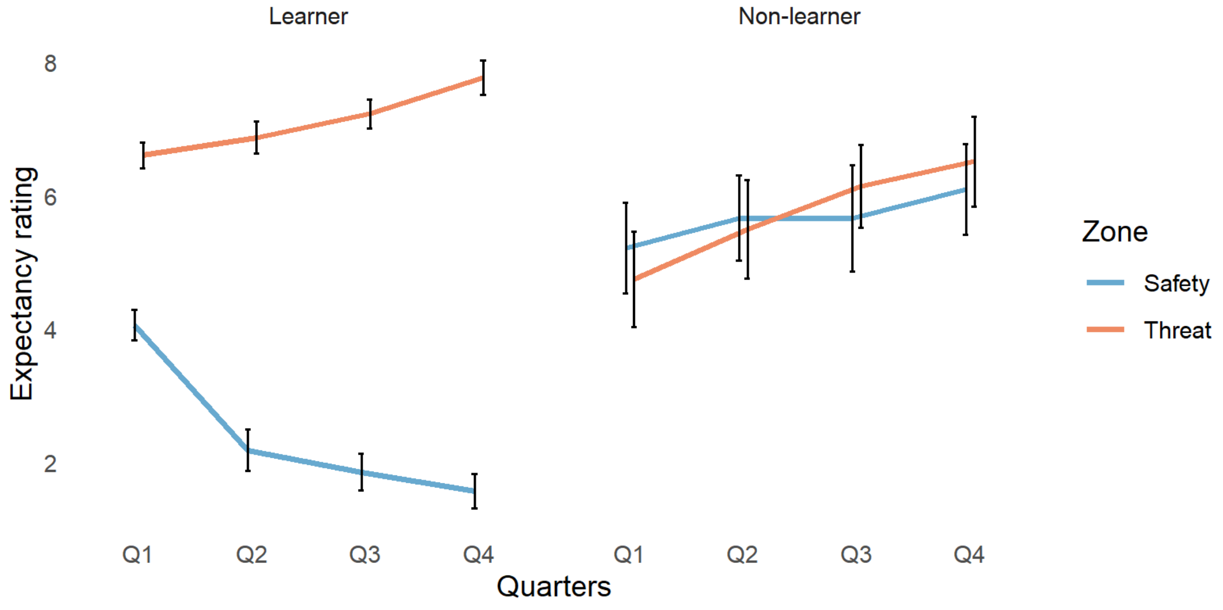

Supplementary Figure 11. Changes in expectancy rating across quarters (Q1-4) for learners and non-learners in the safe and dangerous zones of the environment at a 50% reinforcement rate.

To clarify the directionality of the main effects in 60% reinforcement rate (Supplementary Figure 12), we conducted HSD” post hoc analysis. We found that learners rated the expectancy of the shock higher in threat than in the safe zone (p=1x10^{-16}, 95%CI[3.083, 4.237]). In the safe zone, learners rated the expectancy of the shock lower than non-learners (p=2x10^{-14}, 95%CI[1.742, 3.165]). In the safe zone, shock expectancy was lower in the 3rd (p=.025, 95%CI[-2.218, -.084]) and 4th (p=.001, 95%CI[-2.674, -.416]) quarters than in the 1st quarter. Learners rated the expectancy of the shock lower than non-learners in the 3rd (p=.025, 95%CI[-2.469,-.094]) and 4th (p=8x10^{-4}, 95%CI[-3.664,-.598]) quarters. Learners rated the expectancy of the shock lower in the 4th quarter than the 1st (p=1x10^{-4}, 95%CI[-2.702, -.590]) and 2nd (p=.007, 95%CI[-2.336, -.223]). Overall, shock expectancy ratings were lower in the 4th quarter than the 1st (p=7x10^{-4}, 95%CI[-1.8775, -.3862]) and the 2nd (p=.006, 95%CI[-1.708, -.217]). Overall, ratings of the shock expectancy were higher in the dangerous zone than in the safe zone (p=1x10^{-16}, 95%CI[2.414, 3.146]). Overall, learners rated the shock expectancy lower than non-learners (p=8x10^{-8}, 95%CI[-1.522,-.729]).

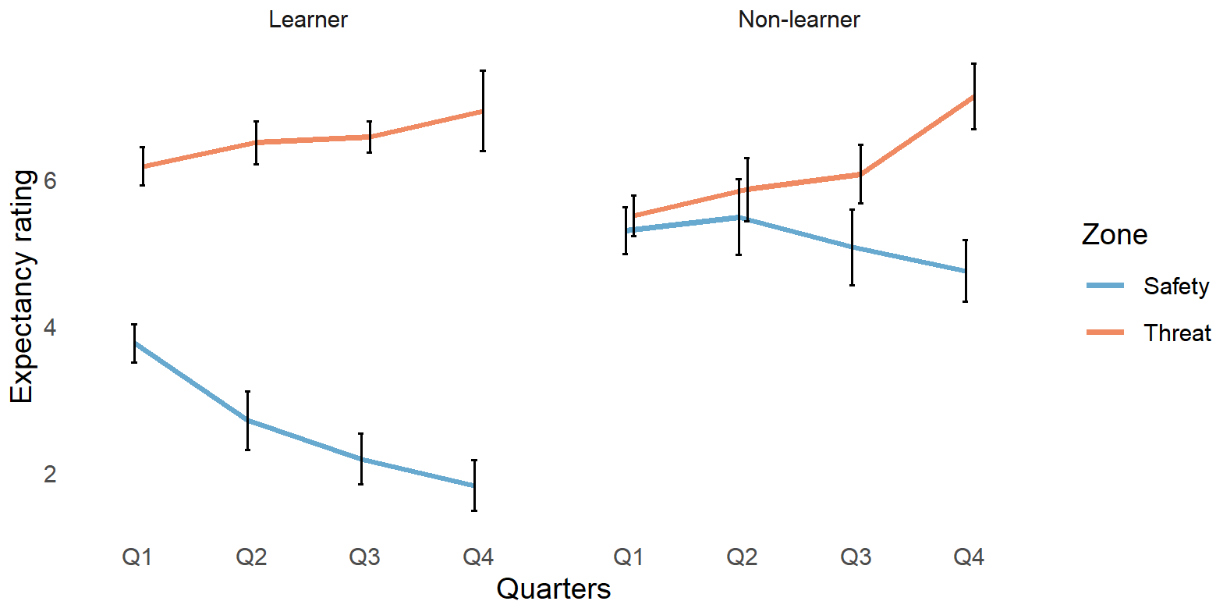

Supplementary Figure 12. Changes in expectancy rating across quarters (Q1-4) for learners and non-learners in the safe and dangerous zones of the environment at a 60% reinforcement rate.
